# Editorial for the “Genetics, Phylogeny, and Evolution of Insects” Special Issue

**DOI:** 10.3390/genes15081000

**Published:** 2024-07-30

**Authors:** Zhiteng Chen

**Affiliations:** School of Grain Science and Technology, Jiangsu University of Science and Technology, Zhenjiang 212004, China; chenzhiteng@just.edu.cn

The rapid advancement of sequencing technologies has revolutionized our understanding of the phylogeny and evolution of insects, enabling researchers to generate extensive molecular data with unprecedented detail. The Special Issue “Genetics, Phylogeny, and Evolution of Insects” brings together 11 original research articles on various advancements of insect genetics and evolution. This Editorial aims to summarize the contributions of the papers published in this Special Issue, highlighting their significance and placing them in a broader context of entomological research.

One of the key themes in this Special Issue is the exploration of mitochondrial genomes, which serve as valuable markers for phylogenetic and genetic studies. Several studies in this collection exemplify this approach, focusing on different insect orders. Regarding Diptera, Bi et al. (2023) [1] sequenced and analyzed the complete mitochondrial genome of *Piophila casei* (Diptera: Piophilidae), a flesh-feeding fly, providing critical information for its genetic structure and phylogenetic position. This study not only aids in understanding the taxonomy and genetics of *P. casei* but also has implications for forensic science and food safety, given the species’ relevance in decaying organic matter and foodstuffs. Similarly, Silva et al. (2024) [2] sequenced the first complete mitochondrial genome of *Orthopodomyia fascipes* (Diptera: Culicidae), revealing a 15,598 bp sequence that supports the monophyly of the family Culicidae and elucidates the evolutionary proximity between tribes Orthopodomyiini and Mansoniini. This study significantly contributes to the taxonomy and evolutionary understanding of the genus *Orthopodomyia* and offers insights into Culicidae, which includes many important vector species.

Regarding Hemiptera, the mitochondrial genomes of three species of *Egeirotrioza* (Hemiptera: Triozidae) were sequenced and analyzed by Aishan et al. (2024) [3], highlighting their evolutionary rates and phylogenetic relationships within Triozidae. This research provides valuable genomic information for the study of psyllid species and underscores the importance of mitochondrial data in resolving phylogenetic controversies. Zhu et al. (2023) [4] sequenced the mitochondrial genomes of three bamboo pests, namely *Notobitus meleagris*, *Macropes harringtonae*, and *Homoeocerus bipunctatus* (Hemiptera: Heteroptera), offering detailed insights into their genetic makeup and phylogenetic placement. This work improves the existing database of bamboo pests and supports the development of rapid identification techniques and pest control strategies.

For Coleoptera, Wang et al. (2023) [5] sequenced and compared the mitochondrial genomes of three tenebrionid species, revealing conserved genomic features and unique tRNA-like structures. Their study supports the monophyly of several subfamilies within Tenebrionidae and provides insights into the evolutionary and functional roles of tRNA-like sequences in these beetles. Li et al. (2023) [6] characterized the mitochondrial genome of *Luperomorpha xanthodera* (Coleoptera: Chrysomelidae: Galerucinae), revealing significant phylogenetic relationships within the family Chrysomelidae. This research contributes to resolving the classification status of *Luperomorpha* and understanding the evolutionary dynamics within the subfamily Galerucinae. Moreover, Yu et al. (2023) [7] sequenced the mitochondrial genomes of four species within the curculionid tribe Scolytoplatypodini, providing detailed phylogenetic analyses and supporting the monophyly of this group. This study enhances our understanding of the evolutionary relationships among scolytine beetles and offers a foundation for further research into their genetic diversity.

Regarding genetics, Liu et al. (2023) [8] explored the genetic diversity and migration patterns of the white-backed planthopper (WBPH) across Myanmar and Yunnan Province. Analyzing the mitochondrial DNA COI genes from 416 individuals, they identified 43 haplotypes, with a high gene flow being indicated by two common haplotypes shared across all populations. This study deduced that WBPH populations in Yunnan primarily migrated from northern and northeastern Myanmar. These findings contribute to the establishment of sustainable management strategies for this significant rice pest in Southeast Asia.

This Special Issue also includes significant contributions from transcriptomic and endosymbiont research, which provide deeper insights into insect biology and ecology. A comparative transcriptomic analysis of the rice leaf folder *Cnaphalocrocis medinalis* (Lepidoptera: Pyralidae) by Du et al. (2023) [9] revealed distinct chemosensory gene expression profiles between larvae and adults. This study highlights the specialized functions of these genes at different developmental stages and suggests potential molecular targets for pest management strategies. The research of Wang et al. (2023) [10] on the gut microbiota of the fall armyworm *Spodoptera frugiperda* (Lepidoptera: Noctuidae) demonstrated that its feeding on various host plants has significant effects on the structure and diversity of its gut bacterial community. These findings underscore the role of the gut microbiota in the adaptive evolution of this major agricultural pest and suggest new avenues for biological control. The study of Corpuz et al. (2023) [11] on *Wolbachia* endosymbionts in Hawaiian Drosophilidae documented the transmission patterns and strain diversity within this adaptive radiation. Their research highlights the complex interactions between *Wolbachia* and their hosts and suggests potential applications in conservation breeding programs for endangered species.

In conclusion, the articles included in this Special Issue entitled “Genetics, Phylogeny, and Evolution of Insects” cover a diverse array of topics and provide comprehensive insights to direct future research in insect genetics, phylogeny, and evolution. The extensive utilization of mitochondrial genomes, transcriptomic analyses, and studies on endosymbionts underscores the complexity and diversity of insect species. These findings highlight the importance of investigating insects not only at the molecular level, but also within the broader context of their ecological interactions and evolutionary histories. The findings presented in this Special Issue both advance our scientific knowledge and have practical implications for pest management, conservation, and biodiversity studies. We anticipate that this Special Issue will inspire further research, leading to a deeper understanding and innovative approaches in the study of insect biology and evolution.
